# Bacteriophage-based control of *Pseudomonas* spp.: research trends and biotechnological challenges

**DOI:** 10.1007/s12223-026-01458-6

**Published:** 2026-03-16

**Authors:** Alice Chiapetti Bolsan, Allan Moreira Terrabuio, Thiago Edwiges, Marina Celant De Prá, Naiana Cristine Gabiatti

**Affiliations:** 1 Programa de Pós Graduação em Sustentabilidade Ambiental Urbana (PPGSAU), Universidade Tecnológica Federal do Paraná, Curitiba, 81280-340 PR Brazil; 2 Licenciatura em Biologia e Biotecnologia - Instituto Politécnico de Bragança, Bragança, 5300-253 Portugal; 3 Programa de Pós Graduação em Biotecnologia (PPGBIOTEC), Universidade Tecnológica Federal do Paraná, Dois Vizinhos, 85660-000 PR Brazil

**Keywords:** Antimicrobial resistance, Biocontrol, Bioproducts, Phage therapy

## Abstract

The bacterial genus *Pseudomonas* is highly diverse, exhibiting broad ecological distribution and significant relevance to human health, agriculture, and the food industry. Several species are recognized as opportunistic human pathogens or phytopathogens and are increasingly associated with microbial resistance, biofilm formation, and persistent contamination. In this context, bacteriophages are promising biotechnological tools for controlling *Pseudomonas* spp. Across multiple sectors, offering high specificity, efficacy, and serving as an environmentally safe alternative to conventional antimicrobials. In this review, we conducted a critical narrative analysis of studies published between 2020 and 2024 addressing the application of bacteriophages against *Pseudomonas* species. Based on bibliometric indicators from the Web of Science database, 250 articles were selected after manual refinement and analyzed to identify publication trends, target species, and application areas. Most studies focused on *Pseudomonas aeruginosa*, primarily in the context of phage therapy for infections caused by multidrug-resistant strains, followed by applications in agriculture and the food industry to control *Pseudomonas syringae* and *Pseudomonas fluorescens*. Keywords co-occurrence analysis revealed trends centered on phage therapy, biofilm control, antimicrobial resistance, genomics, and sustainable biocontrol strategies. Despite the growing body of research, the analyzed studies highlight significant challenges in translating laboratory-scale findings into scalable, commercially viable applications, mainly due to regulatory hurdles to production and standardization. Overall, this review underscores the increasing scientific interest in bacteriophages as biocontrol agents against *Pseudomonas* spp. and discusses prospects for integrating these technologies into healthcare, agriculture, and industrial applications.

## Introduction

*Pseudomonas* spp. are widely distributed and perform relevant ecological functions with biotechnological applications; several species are commensals or pathogens for humans, animals, and plants (Lalucat et al. 2022). Some *Pseudomonas* species are among the primary opportunistic pathogens affecting humans in medicine. These include *P. aeruginosa*, *P. putida*, *P. cepacia*, *P. stutzeri*, *P. maltophilia*, and *P. putrefaciens* (Halabi et al. 2019; Elbehiry et al. 2022; Mokhtar et al. 2022; Sanya et al. 2023). *Pseudomonas* phytopathogens, such as *P. syringae*, *P. cichorii*, and *P. fluorescens*, cause diseases in a variety of crops, and symptoms include cankers, leaf and stem spots, late blight, soft rot, and galls (Mota et al. 2021; Córdova et al. 2023; Gálvez et al. 2024). Species within this genus exhibit remarkable metabolic and genomic adaptability, which contributes to their persistence across diverse environments (De Smet et al. 2017; da Cruz Nizer et al. 2021).

The growing use of antibiotics and the resistance acquired by these bacteria have made eradicating pathogenic species of the genus challenging. Bacteriophages have become attractive in this scenario, emerging as a biocontrol tool for these species. As part of the group of priority pathogens associated with healthcare-associated infections and multidrug resistance, the genus *Pseudomonas* contributes significantly to treatment failure and prolonged hospitalization (Alharbi et al. 2025). This challenge is intensified by intrinsic resistance mechanisms, rapid acquisition of additional resistance determinants, and a strong ability to form biofilms, which together limit the long-term effectiveness of conventional antimicrobial therapies (Pang et al. 2019; Elfadadny et al. 2024).

It should be noted that *Pseudomonas* is a strategic target for phage intervention when compared to other bacterial genera. This is due to the convergence of adaptive characteristics and its widespread presence in clinical, agricultural, and food-processing environments, underscoring the need for alternative antimicrobial strategies specifically tailored to this genus (Saati-Santamaría et al. 2022; Alattas et al. 2024; Alexandre et al. 2025).

The ecological diversity and ubiquity of *Pseudomonas* spp. are mirrored by their bacteriophages, which are broadly distributed and readily isolated from a wide range of environments, supporting their investigation and development as biocontrol agents (Rafiq et al. 2022; Hashemi Shahraki et al. 2023; Kamtaeja et al. 2024; Zaer-Anaqz et al. 2024; Usman and Christina 2024). The use of phages in the current context of antimicrobial resistance offers functional advantages to the *Pseudomonas* genus, including host specificity, self-replication at the site of infection, and the ability to target bacterial populations associated with biofilms through phage enzymes such as depolymerases and endolysins (Anastassopoulou et al. 2025; Husain et al. 2025). This combination of characteristics makes phage-based control advantageous for *Pseudomonas* when compared to other bacterial genera, especially those in which biofilm composition and intrinsic resistance are less structurally integrated.

Given the expanding body of research on bacteriophages targeting *Pseudomonas* spp., a structured synthesis of application patterns and translational progress is needed. This study presents a critical, application-oriented narrative review supported by bibliometric indicators to contextualize research trends from 2020 to 2024. A curated dataset of publications was systematically analyzed to enable cross-sector comparison across clinical, agricultural, and food-related applications. We evaluated host range and strain specificity, experimental context (in vitro assays, in planta studies, animal models, clinical reports, and field trials), reported efficacy metrics (e.g., log reductions, symptom severity, survival outcomes), and indicators of translational maturity, including formulation strategies, commercial development, and regulatory status. By integrating bibliometric mapping with structured qualitative assessment, this study aims to identify dominant research trajectories, sector-specific bottlenecks, and gaps that may limit the transition from experimental validation to scalable application.

## Bibliometric methodology and data refinement

To understand the research development, a literature search was conducted using the Web of Science Core Collection database. A Topic Search (TS), encompassing titles, abstracts, author keywords, and Keywords Plus, was conducted using the following Boolean query: TS= (bacteriophage and pseudomonas). The use of broad descriptors was intentional to maximize sensitivity and capture the widest possible range of publications on bacteriophage applications targeting the *Pseudomonas* genus. The search was restricted to publications from January 2020 to November 2024. Only peer-reviewed articles published in English were included. Conference proceedings, book chapters, editorials, and notes were excluded.

The initial search yielded 652 records at the time of data collection, with refinement completed on November 5, 2024. A manual screening procedure was performed to ensure relevance for bacteriophage applications targeting *Pseudomonas* spp. Studies focused exclusively on phage genomics without applied context, studies involving non-*Pseudomonas* hosts, and articles classified as research papers but consisting only of narrative reviews of the literature without experimental or applied data were excluded. After using the inclusion and exclusion criteria, 250 articles were retained for the final analysis dataset.

**Fig. 1:**
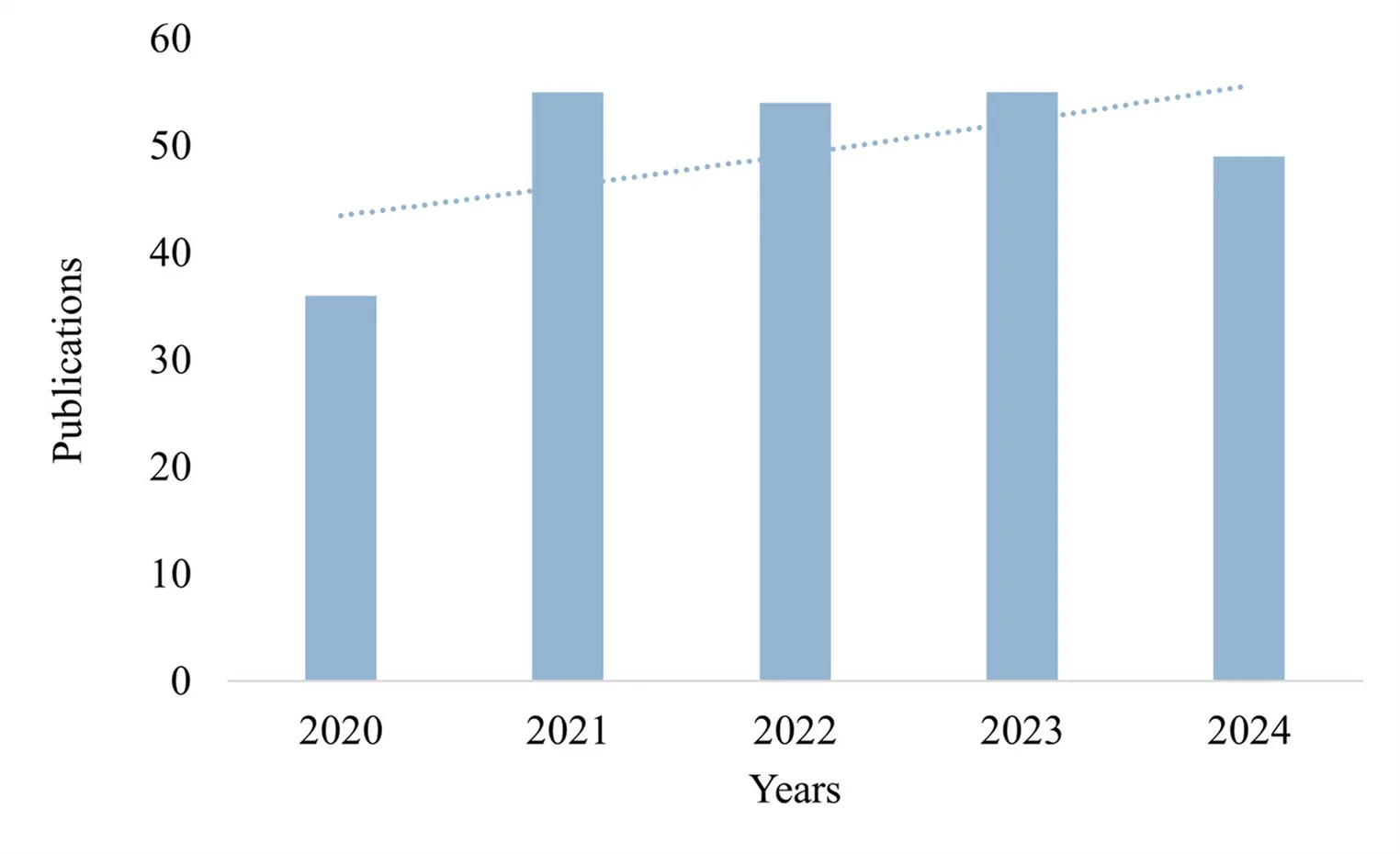
Annual distribution of publications included in the final dataset (2020–2024), with bars showing yearly counts and a dotted trendline indicating the increasing research trend on bacteriophages and *Pseudomonas*

The annual distribution (Fig. 1) was used to illustrate the dynamics of publications during the selected period, providing contextual support for the subsequent sectoral analysis.

In addition to temporal distribution, the dataset revealed a concentration of publications originating mainly from research groups based in the United States, China, and Australia, reflecting strong research activity in clinical microbiology, antimicrobial resistance, and applied phage therapy. The most recurrent institutional affiliations included the University of Sydney (10 publications), the Egyptian Knowledge Bank (9 publications), and Queen Astrid Military Hospital (9 publications), indicating the prominent role of major university systems and research centers specializing in infectious diseases and biological control strategies.

**Fig. 2:**
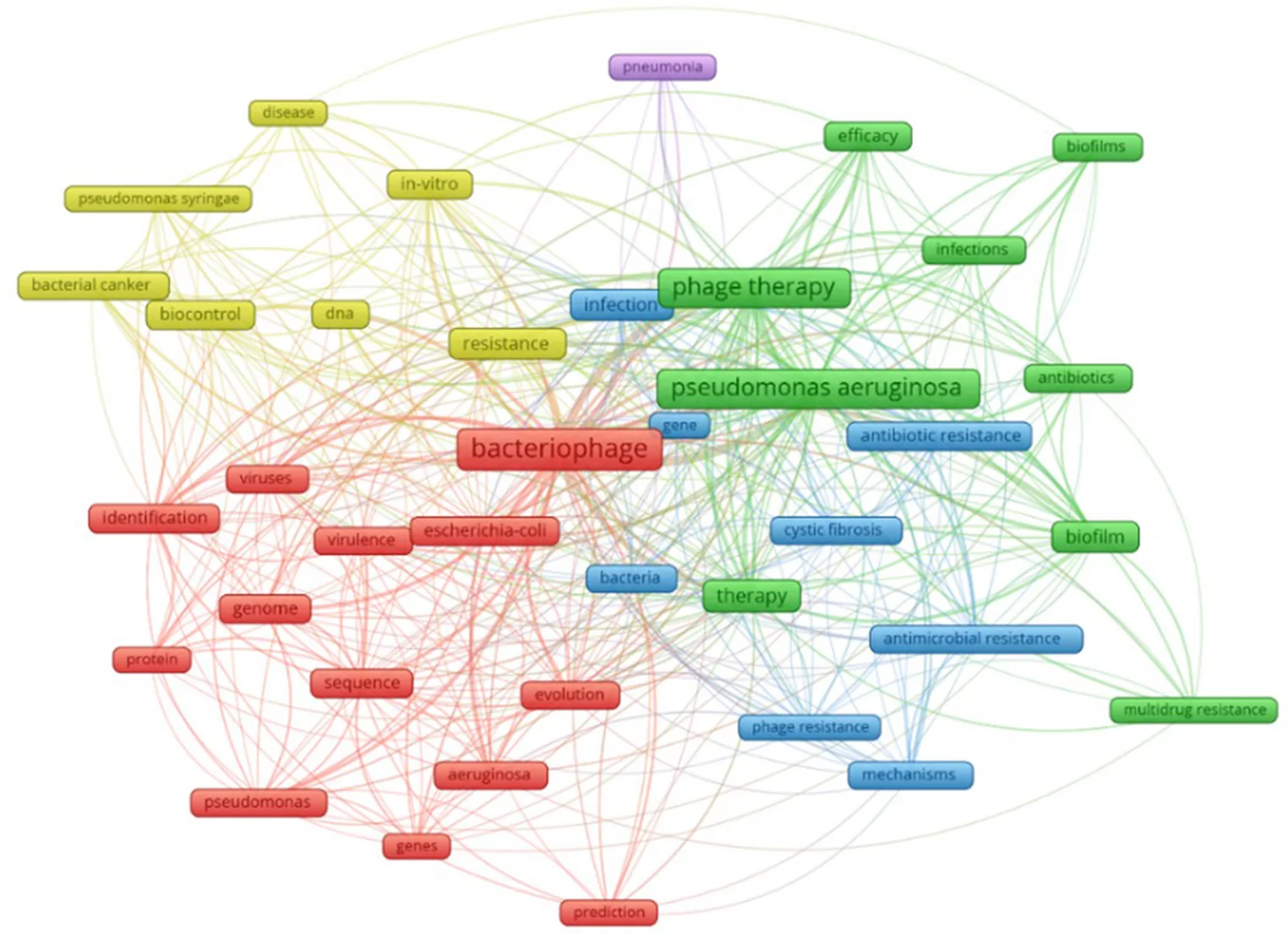
Keyword co-occurrence network of the 250 publications analyzed

The publications were predominantly distributed across journals classified under the Web of Science research categories of Microbiology, Virology, Pharmacology & Pharmacy, and Biotechnology & Applied Microbiology, highlighting the interdisciplinary nature of phage research targeting *Pseudomonas* spp. The growing number of publications over the last decade correlates with the rise of the antibiotic crisis, reflecting the scientific community’s increasing interest in alternative solutions, such as bacteriophage therapy. Analyzing the keywords used in the 250 publications using the VOSviewer software version 1.6.19 (van Eck and Waltman 2010) made it possible to observe some trends and applications already under study (Fig. 2).

Analysis of the keywords reveals a diverse yet interconnected set of research topics that can be grouped into four dominant clusters. The most notable group focuses on clinical phage therapy, strongly associated with terms such as “phage therapy,” “biofilm,” “antibiotic resistance,” and “multidrug resistance,” reflecting the growing focus on bacteriophages as alternative strategies to combat infections, particularly those caused by antimicrobial-resistant *Pseudomonas aeruginosa*.

A second group concerns the biocontrol of plant diseases, as evidenced by keywords such as “*Pseudomonas syringae*” and “bacterial canker,” highlighting the relevance of phage-based approaches in agricultural disease management. A third thematic direction involves food preservation and spoilage control, linked to biofilm reduction and microbial load management in food systems. In addition, there are terms related to genomics and molecular biology (“genome,” “sequence,” and “genes”), reflecting the importance of genomic and molecular studies in understanding phage-host dynamics and supporting applied developments in all sectors.

Given the growing advances in bacteriophage research, the thematic groups provide a conceptual framework for the discussion in the following sections, allowing for the comparative analysis of trends, technological maturity, and challenges in using bacteriophages to control bacteria of the genus *Pseudomonas* in health, agriculture, and food.

## Bacteriophage application

### Bacteriophage vs. *Pseudomonas* in healthcare

**Fig. 3:**
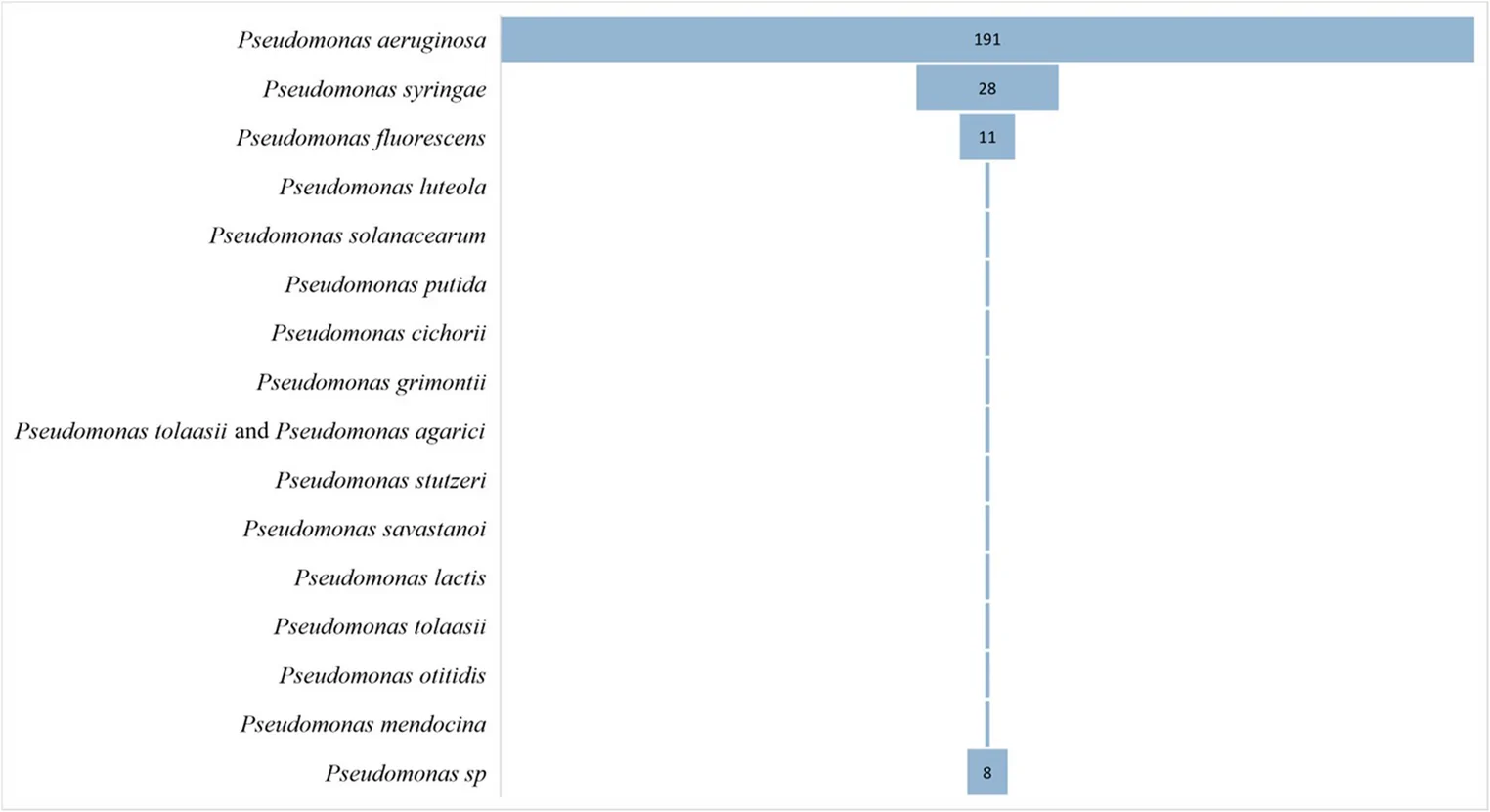
Distribution of *Pseudomonas* species identified in the 250 publications analyzed

Bacteriophages specifically targeting this genus were first identified in the mid-20th century (Chegini et al. 2020). As indicated by the clinical therapy thematic cluster in Fig. 2, a substantial portion of recent scientific output focuses on multidrug-resistant *Pseudomonas aeruginosa* infections and biofilm-associated clinical conditions. The increasing incidence of nosocomial infections, combined with the high levels of antibiotic resistance exhibited by *P. aeruginosa*, has intensified interest in phage-based strategies to control its growth. Consistent with this trend, *P. aeruginosa* was the most extensively studied species within the refined dataset, accounting for 191 of the 250 analyzed publications (Fig. 3). The predominance (76% of the studies analyzed) reflects the clinical significance associated with this species and the focus of research on its control, especially in hospital settings.

According to a report by the US Centers for Disease Control and Prevention, it is estimated that approximately 32,600 healthcare-associated infections caused by *P. aeruginosa* occur in the United States each year, and 8% of these infections are multidrug-resistant (MDR), with around 270 deaths per year attributed to such infections, (Centers for Disease Control and Prevention 2019). These data reflect the clinical importance of this representative species within the genus and its adaptive capacity in the face of antimicrobial resistance.

Furthermore, it is predicted that drug resistance will result in 10 million premature deaths annually worldwide and cost an estimated loss of US$100 trillion (Lauman and Dennis 2021). Globally, the increase in publications focusing on the use of bacteriophages to control *Pseudomonas* in different areas reflects a shift toward alternative interventions targeting multidrug-resistant bacterial genera.

The resistance acquired by this species is both intrinsic and acquired from other microorganisms. Mechanisms include a decrease in the permeability of the outer membrane, which prevents the drug from being delivered into the cell, excessive activity of efflux pumps, which transport the antibiotic out of the cell, genes or mutations in antibiotic resistance genes, which act to control the passive diffusion of the drug across the outer membrane (Nawaz et al. 2024). This species thrives in a wide range of environments due to its ability to adhere to diverse surfaces, facilitated by structures such as pili, fimbriae, and its capacity to form biofilms (Shafigh Kheljan et al. 2023).

Bacteriophages present significant advantages over antibiotics in controlling biofilm-associated infections. While antibiotics mainly target bacteria at the biofilm surface, bacteriophages can penetrate deeper layers and disrupt the biofilm matrix through phage-derived or host-induced enzymes. In addition, bacteriophages can infect and eliminate persister cells, even after reactivation (Chegini et al. 2020; Gliźniewicz et al. 2024).

Given the health issues associated with this strain of *Pseudomonas*, numerous in vivo studies have been conducted, with some demonstrating the efficacy of phage therapy in humans. Analyzing the selected articles, it is evident that most cases occur in in vivo animal models and in compassionate use in humans, with efficacy assessed primarily through clinical resolution, microbiological elimination, and patient follow-up.

Of the articles that focused on the use of phages to control *Pseudomonas* in the healthcare sector, 53 carried out in vivo tests. The experiments included applications to animals (mainly rats) and to humans. Considering the total set of 250 articles, these 53 in vivo studies represent approximately 21%, indicating that most remain in the laboratory or preclinical phase.

Table 1 presents some of the studies that have applied phage therapy directly in humans. The use of compassionate treatment, joint application with antibiotics, and heterogeneity in routes of administration indicate that phage therapy against *P. aeruginosa* remains at an early stage of clinical maturity, with few controlled clinical trials available.

**Table 1:** Summary of reported human clinical cases using bacteriophage therapy against *Pseudomonas aeruginosa* and the results

Patient profile	Resistance profile	Infection type	Route of Administration	Phage formulation	Combined with an Antibiotic	Clinical Outcome	Follow-up duration	Reference
68-year-old male; bronchopleural fistula; history of pulmonary tuberculosis.	Carbapenem-resistant	Pneumonia with empyema	Nebulization and intrapleural	Two-lytic-phage cocktail	Yes	Clinical recovery and eradication reported	Radiological evaluation at day 39 post-therapy	(Chen et al. 2022)
57-year-old male; double lung transplant recipient.	Extensively drug-resistant (XDR)	Chronic bronchopulmonary infection	Inhalation + oral administration	Phage therapy (with IgM/IgA immunoglobulins)	Yes	No further infections after the second cycle; fatal respiratory failure	Not specified (≥ 1 year post-transplant documented)	(Levêque et al. 2023)
Pediatric patient; post-liver transplantation	Drug-resistant (post-transplant)	Sepsis with liver abscess	Intravenous infusion	Phage cocktail	Yes	Clinical and microbiological improvement; successful. retransplantation	Therapy duration 86 days (follow-up not clearly specified)	(Van Nieuwenhuyse et al. 2022)
Two patients (6 and 26 years old), both with cystic fibrosis.	Pan-drug-resistant (PDR)	Chronic pulmonary infection	Inhalation/nebulization	Phage therapy	Yes	Mixed outcomes (improvement; death due to disease progression)	5–6 months	(Hahn et al. 2023)
74-year-old patient	Pandrug-resistant (PDR)	Spinal infection with abscess	Local + intravenous	Phage cocktail	Yes	Resolved	21 months	(Ferry et al. 2022)
21-year-old man	Multidrug-resistant (MDR)	Femoral osteomyelitis	Local + intravenous	Phage cocktail	Yes	Eradication of infection, amputation avoidance.	9 months	(Racenis et al. 2022)

In healthcare applications, *P. aeruginosa* emerges as the most clinically relevant species. Preclinical trials in animal models, along with early-stage human studies, have shown promising results, underscoring the potential of bacteriophages to combat antibiotic-resistant infections.

It is essential to highlight that host variation among clinical isolates and the possibility of phage-resistant mutants emerging represent biological constraints that may affect long-term therapeutic consistency. In addition, uncertainty regarding regulations, the logistics of individualized production, and the need for rapid matching between phages and bacteria pose operational and economic challenges for large-scale implementation. Nevertheless, consolidating this therapeutic approach will require expanding controlled clinical trials to enable robust assessments of efficacy and safety, as well as standardizing treatment protocols.

### Bacteriophage vs. *Pseudomonas* in the food industry

In the food industry, *Pseudomonas* spp. are widely distributed and can be found in dairy products, meat products, vegetables, and even water (Ruiz-Roldán et al. 2021; Li et al. 2024; Chang et al. 2024). Among the articles we analyzed in the food sector, studies are predominantly conducted in vitro or under controlled storage conditions, with efficacy typically measured by logarithmic reductions in CFU and biofilm. The pattern observed in most investigations remains at the laboratory or pilot scale, with limited evaluation under full industrial processing conditions.

Their presence is also associated with their ability to form biofilms on processing surfaces, which increases their persistence and resistance to cleaning and sanitization procedures (Li et al. 2023). Among these species, *P. fluorescens* (the most studied for food control, according to 250 articles) is primarily found in milk and dairy products, while *P. aeruginosa* is more prevalent in meat and seafood (Alexandre et al. 2025). Species such as *P. putida* are commonly associated with meat spoilage (Papadopoulou et al. 2020). The thematic cluster related to food preservation and spoilage control, shown in Fig. 2, highlights biofilm reduction and microbial load control as dominant research directions in food-related applications.

The psychrotrophic characteristic of *Pseudomonas* spp. allows them to survive at low temperatures, even in refrigerated environments, and they are also capable of producing proteolytic and lipolytic enzymes that compromise food quality (Ávila et al. 2025). Understanding the ecology and biofilm-forming ability of these species is crucial to designing effective control strategies in the food processing industry. The persistence of *Pseudomonas* spp. in food matrices is explained by the above characteristics and justifies the focus on biofilm disruption and microbial load reduction in phage-based strategies.

Conventional methods for eradicating strains include the use of chemical disinfectants; however, these can prove insufficient and raise concerns about residues and bacterial resistance (Karpiński et al. 2025). As a result, phage-based approaches are emerging to improve food safety and extend shelf life.

In the food industry, bacteriophages can be applied directly to animals and plants to eliminate the likelihood of bacterial infection and disease, as a preventative method before the raw material enters the industry for processing, or directly to the food to preserve the product (Cristobal-Cueto et al. 2021).

Satisfactory results were reported when a bacteriophage specific to *P. aeruginosa* was applied to a biofilm; after 8 h of treatment, more than 95% of the biofilm had been reduced. When applied to control the same species in milk and beef after 12 and 24 h of treatment, the bacterial reduction was 68.34% and 92%, respectively, with complete reduction achieved after 36 h (Yang et al. 2025). The effectiveness of phage treatment may depend on exposure time and be influenced by storage conditions, reinforcing the importance of timing of application and optimization of dosage.

When a bacteriophage was used to control *P. fluorescens* in milk, a significant reduction in bacterial growth was observed at both 7 °C and 28 °C, indicating that the effectiveness of the phage varied depending on the multiplicity of infection (MOI). In addition, the use of the phage resulted in less protein degradation and less acidification of the milk (Yuan et al. 2025). A phage cocktail was applied against *P. fluorescens*, *P. tolaasii*, and *P. rhodesiae* in milk after storage at 4 °C for 5 days. The application resulted in reductions of up to 4.09 log CFU/mL in whole dairy and 5.29 log CFU/mL in skim milk (Tayyarcan and Boyaci 2023). The difference in control efficacy in whole milk and skim milk samples is likely associated with fat composition and content, which influences the interaction and diffusion between phages and their hosts (Khan et al. 2023).

The ability of *P. aeruginosa* to form biofilms on industrial surfaces such as stainless steel poses a challenge for the food processing industry in terms of hygiene and microbial control. Phages were applied to biofilms that had grown for 24 h, originating from *P. aeruginosa* strains. The results showed that the treatment reduced bacteria in biofilms formed on the stainless steel surface by 1.7 log CFU/cm² compared to untreated biofilms (Magin et al. 2019). The moderate decrease in log suggests that biofilm composition and surface properties influence the likelihood of complete eradication, with possible solutions including combined treatment with other antimicrobial products or repeated treatments.

In tests on chicken meat contaminated with *P. aeruginosa*, the application of two phages reduced the bacterial load when the meat was stored at 4 °C. In addition to acting as biological preservatives in the food itself, the lytic activity was maintained under real storage conditions, demonstrating the potential of these viruses for use in the food industry (Yilmaz et al. 2024).

In pasteurized milk and fish food matrices, the use of a phage satisfactorily reduced the bacterial concentrations of *P. fluorescens*. In milk stored at 25 °C for 48 h, there was a reduction of 2.71 log CFU/mL and 2.84 log CFU/mL at multiplicity of infection of 100 and 1000, respectively, representing an approximate reduction of 99.81% and 99.86% in bacterial load. When applied to the fish matrix at 25 °C for 24 h, there was a decrease of 1.28 CFU/mL (MOI 100) and 2.64 CFU/mL (MOI 1000), corresponding to reductions of 94.75% and 99.77% in the bacterial population (Yan et al. 2023). These findings suggest that phage performance can vary depending on the food matrix and multiplicity of infection, emphasizing the need for tailored application strategies in different products.

Overall, available evidence indicates that bacteriophages can significantly reduce bacterial load and biofilm formation in various food matrices under controlled conditions. In addition, they contribute to food safety by targeting pathogenic bacteria without altering the food’s taste, texture, or nutritional value. Furthermore, they help extend the product’s shelf life by reducing bacterial load (Ranveer et al. 2024). However, environmental stability (temperature fluctuations, pH), matrix composition (fat and protein content) can influence performance under real-world conditions; in addition, regulatory approval pathways, consumer acceptance, and large-scale production costs remain decisive factors for industrial implementation.

**Fig. 4:**
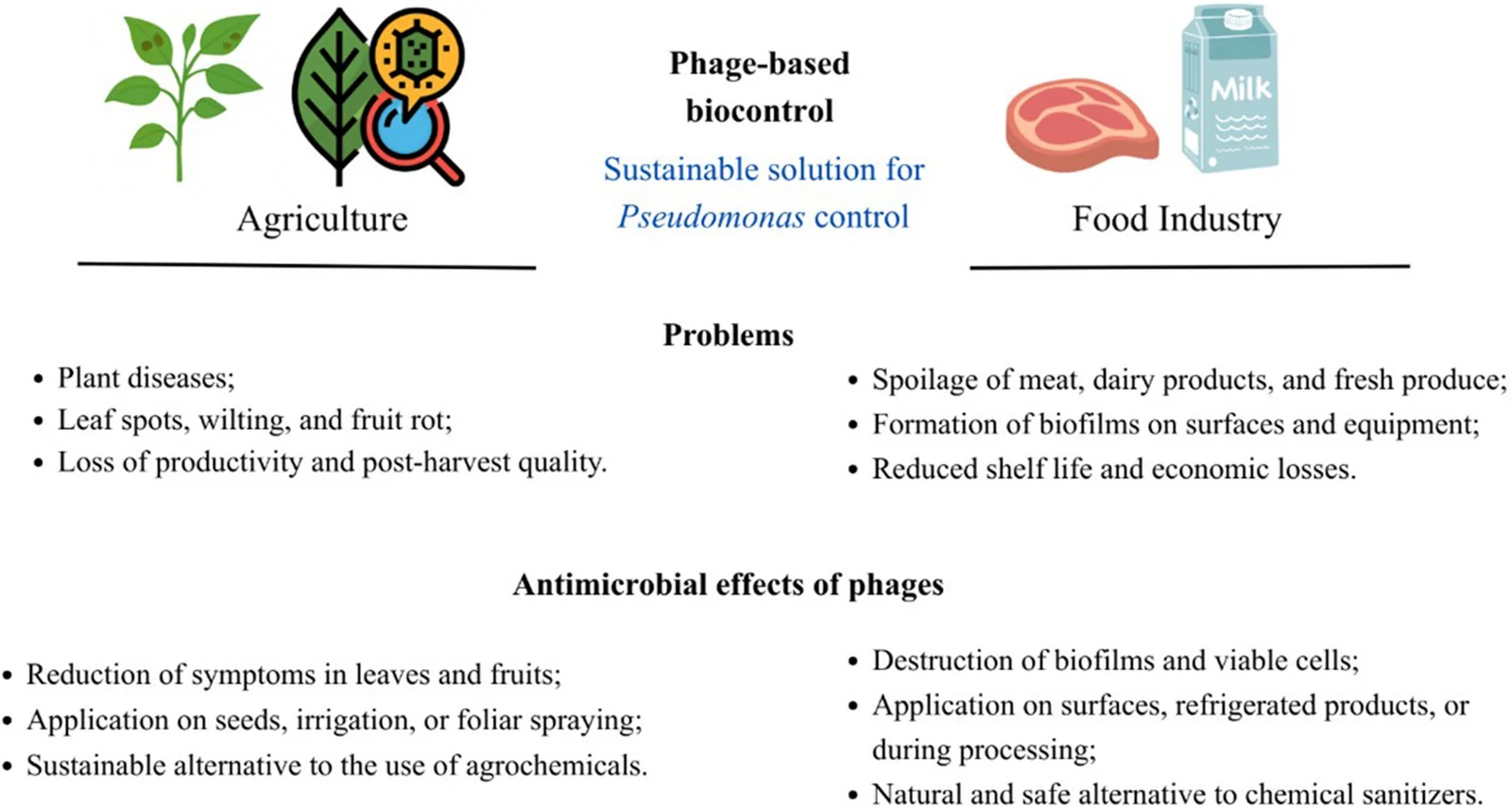
Applications of bacteriophage biocontrol in the control of *Pseudomonas* in agriculture and the food industry

Bacteriophages act as a control tool at the beginning of the production chain, since *Pseudomonas* contamination can occur in the field, affecting the quality of the raw material that reaches the food industry. As illustrated in Fig. 4, the application of these agents across both agriculture and the food industry represents an integrated biosafety strategy that contributes to a more sustainable, safe, and efficient production system.

### Bacteriophage vs. *Pseudomonas* in agriculture

Bacterial diseases in plants cause economic losses and pose a significant challenge to global agricultural productivity. These diseases reduce production and affect food security. Post-harvest losses also contribute to resource waste, soil degradation, and deforestation (Kaloudis et al. 2025).

In a scenario where sustainable methods are increasingly sought after, sustainable agriculture aims to employ practices that result in disease-free plants and animals, thereby providing safe food and minimizing the environmental impact of agricultural activities (Svircev et al. 2018). The main strains of *Pseudomonas* that cause disease in agriculture are: *P. syringae*, *P. savastanoi*, *P. corrugata*, and *P. viridiflava* (Mehmood et al. 2023). The *Pseudomonas* genus can cause significant damage to crops, presenting symptoms such as leaf spots, wilting, burning, and rotting. These phytopathogens can spread rapidly through plant tissues, contaminated soil, or water sources, making their control challenging with conventional chemicals alone (Giovanardi et al. 2025).

In agricultural applications, efficacy is typically evaluated in planta or in the field, with results measured as reductions in disease incidence, symptom severity, or epiphytic bacterial load. For control of pathogenic bacteria in agriculture, phages are applied to plant crops by spraying or directly into the soil, reducing the incidence and severity of disease. This procedure is environmentally friendly, as it does not use chemical pesticides and does not affect beneficial microorganisms in the soil (Thi Trang Nhung et al. 2025).

*Pseudomonas syringae* strains are responsible for a large number of diseases in agricultural plants, and the lack of methods or failure of existing ones redirects research toward the use of phages as a biocontrol agent (Svircev et al. 2018). Since 2008, the kiwifruit industry has been significantly impacted by *P. syringae* pv. *actinidiae*, the bacterium responsible for bacterial canker in kiwifruit. Commonly used treatments are based on copper and antibiotics, but challenges arise related to resistance and soil contamination (McCann et al. 2017).

In a study conducted in commercial kiwi orchards, a mixture of phages effectively reduced leaf damage associated with *P. syringae* pv. *actinidiae* and decreased the epiphytic bacterial population load. In the first harvest, reductions of up to 40–45% in bacterial load were observed after 60 days. In both orchards, during the first season, bacteriophages also outperformed copper-based and antibiotic treatments (Sanhueza et al. 2025). The reductions observed in the field indicate that phage use can surpass the efficacy of conventional treatments with copper and antibiotics under commercial orchard conditions, suggesting practical viability beyond laboratory tests.

The application of a commercially available bacteriophage directly to kiwi leaves inoculated with *P. syringae* pv. *Actinidiae* resulted in reductions of 1.1 and 1.8 log CFU/mL in two strains after 24 h of treatment. These results indicate that phage lytic activity remains effective even under more complex environmental conditions (Pinheiro et al. 2020).

In an experimental study evaluating phage therapy against bacterial canker caused by *P. syringae* pv. *syringae* on apricot leaves showed a significant reduction in infection levels over 24 to 72 h, with an 82% reduction in bacterial count after phage application. Visually, a decrease in typical symptoms of the disease was observed, including changes in coloration, darkening, and the appearance of perforated leaves, which were also present in leaves treated only with the bacterium (Selcuk et al. 2024). The potential use of phages extends beyond antimicrobial agents to include agents capable of reducing agronomic impact, as they simultaneously reduce bacterial load and the severity of visible symptoms.

The application of a phage to tomato plants infected with *P. syringae* pv. *tomato*, through root irrigation and foliar spraying, reduced the bacterial population by up to 60 times through root treatment, while when applied by foliar spraying, it reduced the severity of the disease (bacterial spot) by approximately six times. Another factor observed was the activation of some plant defense genes, genes associated with protective responses in uninfected tissues and defense against biotic stresses, indicating that the phage not only controls the pathogen but also stimulates the plant host’s immune mechanisms (Skliros et al. 2023). The observed effect, both in terms of direct bacterial control and stimulation of plant defense responses, shows that phages can act through both lytic activity and indirect mechanisms mediated by the host.

Soybeans are a vital crop worldwide, serving as a crucial source of vegetable protein for humans and livestock globally. *Pseudomonas savastanoi* pv. *glycinea* is the causative agent of bacterial blight in this crop, where it is considered the most destructive of soybean diseases, reducing yields by up to 40% (Jagtap et al. 2012).

When a phage was applied to soybean leaves, it significantly reduced infection caused by *P. savastanoi* pv. *glycinea*, decreasing the disease by about 52% (phage concentration at 10⁷ PFU/mL). In the treatment of seeds infected with the pathogenic strain, the phage demonstrated a biological efficiency of 59.7% (disease incidence), resulting in a reduction in infected seedlings and a lower disease development rate (Tarakanov et al. 2022). It should be noted that phages have potential applications at both the leaf level and in seed treatment, thereby expanding their applicability across different stages of agricultural production.

In trials on coffee seedlings inoculated with *P. coronafaciens* pv. *garcae*, the application of two lytic phages encapsulated in chitosan-coated calcium alginate nanoparticles was effective in reducing the severity of bacterial canker. The application occurred before and after bacterial inoculation, resulting in a reduction in disease severity between 66.83% and 83.37% compared to control plants (Silva et al. 2025). The application of phages via encapsulation delivery suggests that formulation technologies can enhance the stability and persistence of phages under field conditions, thereby addressing one of the main limitations to environmental exposure.

Bacteriophages are a promising and efficient strategy for controlling different strains of *Pseudomonas* in vivo. In addition to reducing bacterial load and symptom severity, they can induce defense responses in the plant host, making them potential sustainable biotechnological tools for managing agricultural diseases.

However, environmental instability, driven by UV radiation, temperature fluctuations, and desiccation, can reduce phage persistence in open-field applications. Another relevant factor for the broader implementation of this biological control using phages is the issue of regulatory integration across agricultural systems and its adoption by farmers. Thus, although field performance is increasingly documented, sustainable integration into agricultural practices will depend on optimized formulations and standardized regulatory frameworks.

## Bacteriophage-based bioproducts

Bacteriophage-based bioproducts can be formulated in a variety of ways, including lytic phages, phage cocktails, enzymes such as depolymerases and endolysins derived from phages(Venkataraman et al. 2025; Husain et al. 2025) and various formulations, such as sprays, gels, and microencapsulations (Bolsan et al. 2024). The mode of action may involve direct lysis of the bacterial culture, degradation of biofilms (Martinho et al. 2024), and, in some cases, combined use with antibiotics(Anastassopoulou et al. 2024) increasing the efficiency of these bioproducts.

Despite the growing number of studies on the use of bacteriophages for *Pseudomonas* control, the majority of them are still restricted to laboratory scale and in vitro applications. Consequently, the commercialization of phage-based products targeting *Pseudomonas* remains limited (Table 2), particularly when compared to well-established bacteriophage products for the control of other pathogenic bacteria, such as *Listeria*, *Salmonella*, and *Escherichia coli* (Hyla et al. 2022).

**Table 2:** Commercially available and experimental bacteriophage-based bioproducts targeting *Pseudomonas* spp. across different application sectors

Bacteria	Bioproduct	Sector	Regulatory status	Country	Application	Reference
*A. hydrophila* and *P. fluorescens*	BAFADOR^®^	Veterinary/Aquaculture	Commercially available	Poland	Additive for fish	(Schulz et al. 2019)
*Staphylococcus*, *Streptococcus*, *Pseudomonas*, *Proteus*, and *E. coli*	Pyophage	Human	Approved and sold locally	Georgia, Belgium, and Russia	Treatment of otitis, burns, chronic wounds, and respiratory infections	(Abedon et al. 2011; Zaldastanishvili et al. 2021)
*X.campestris* pv. *vesicatoria* e *P.syringae* pv. *tomato*	Agriphage	Agriulture	Commercially available	USA	Control of phytopathogenic bacteria in plants	(Choudhary et al. 2025)
*Pseudomonas* spp. and other plant pathogens	Biolyse-PB™	Food/Agriculture	Commercially available	United Kingdom	Post-harvest control, washing, and disinfection of vegetables	(Vero et al. 2023)
*P. aeruginosa*	PhageBank^®^ (Adaptive Phage Therapeutics)	Human	Experimental/Compassionate use	USA	Applied in clinical cases of multidrug-resistant infections caused by *P. aeruginosa*	(Onallah et al. 2023b;2023a)
*P. aeruginosa*	Personalized phage cocktail	Human	Compassionate use/Investigational use	Pherecydes Pharma (Romainville, France)	Application in bone infection caused by multidrug-resistant *P. aeruginosa*	(Ferry et al. 2018)
*P. aeruginosa*	PhageBioderm	Human	Commercially available	Eliava Institute - Georgia	Topical treatment of chronic wounds and infected ulcers	(Markoishvili et al. 2002)
*P. aeruginosa*	Personalized phage cocktail	Human	Personalized preparation/Compassionate use	Rutgers University, Texas Tech University, and Yale University	Nebulization to reduce bacterial load in resistant pulmonary infections	(Chan et al. 2025)
*E. coli* and *P.fluorescens*	Alginate film/coating with phages	Food	Experimental/Research stage	Portugal	Coating for application on surfaces or packaging	(Coelho et al. 2025)
*P. syringae*	Seed coating - immersion of seeds in phage suspension	Agriculture	Experimental/Research stage	USA/Japan	Seed coating	(Erdrich et al. 2024)

Commercial maturity is most evident in products for agricultural and food applications, while human applications outside Eastern Europe remain largely restricted to compassionate or investigational use. This contrast highlights the uneven regulatory and technological development of phage-based interventions.

Phage formulations are regulated differently around the world, reflecting each country’s legal and health approaches, which is one of the main limitations to the commercial implementation of these biological agents. The requirements governing the use of phages are more complex and less consolidated when compared to the use of antibiotics (Yang et al. 2023; Fuerst-Wilmes et al. 2025). When isolating a phage or a phage cocktail, a certificate of safety, efficiency, and production must be obtained, which increases the cost and development time of these bioproducts.

In Eastern European countries such as Georgia, Poland, and Russia, phage therapy has been used since its initial discovery (Międzybrodzki et al. 2018). In Georgia and Russia, phage cocktails are marketed as medicines under local regulations and can be purchased without a prescription for human use (Abedon et al. 2011).

In the United States and the European Union, approval processes are more restrictive. In the US, there are currently no phage products commercially approved by the Food and Drug Administration (FDA) for clinical use (Berryhill et al. 2025). In Europe, bacteriophage therapy has been classified as a medicine by the European Medicines Agency since 2011 (Yang et al. 2023). The regulatory differences across countries directly affect the availability, production, and application of phages as bioproducts, underscoring the need for standardization to expand their safe and effective use.

In agriculture, the use of phage products is already well-established. In the USA, the first product registered for controlling bacterial diseases in plants was introduced in 2006, named Agriphage, targeting *Xanthomonas campestris* pv. *vesicatoria* and *P. syringae* pv. *tomato* in peppers and tomatoes (Choudhary et al. 2025).

In the fields of animal health and the food industry, phages were first used in 1919 by Félix d’Hérelle, the discoverer of these organisms. Phage preparations were tested for the prevention of fowl typhoid caused by *Salmonella gallinarum* in poultry (Vandamme and Mortelmans 2019). In the following decades, in Eastern Europe and the USA, the development of regulated commercial products such as ListShield™, EcoShield™, and SalmoFresh™ began, used to control *Listeria monocytogenes*, *Escherichia coli*, and *Salmonella enterica* in food, feed, and processing surfaces, serving as the basis for the growth of phage biotechnology in modern animal health (Garvey 2022).

When analyzing the development of phage products for *Pseudomonas* control, a distinction is made between areas of application. In the field of human health, there are no globally approved products; most are experimental cocktails mainly for *P.aeruginosa*, used in compassionate use protocols (Onallah et al. 2023b).

A common practice in healthcare is the preparation of bacteriophage-based formulations for specific cases, which are not commercial products but are legally authorized for use. In the US, the Center for Innovative Phage Applications and Therapeutics at the University of California, San Diego, produces phage cocktails for patients with *P. aeruginosa* infections, including cases of cystic fibrosis or post-surgical infections (Aslam et al. 2020). In Belgium, Queen Astrid Military Hospital uses phages as medicine under authorization from the Belgian Federal Agency for Medicines and Health Products (FAMHP) (Verbeken and Pirnay 2022). Both examples are approved for master use, meaning they are legally administered but not standardized commercial products.

In animal health, phage formulations are currently in the research and development stage (Antoine et al. 2025). However, agriculture is the only commercial sector with established products for *Pseudomonas*, notably AgriPhage™ formulations designed to control *P. syringae* in tomato and pepper crops (Hoffmann et al. 2025). In the food industry, however, the use of phages is currently limited to the experimental level, primarily focusing on controlling P. fluorescens to reduce food spoilage and *P. aeruginosa* to control biofilms on processing surfaces (Elafify et al. 2024; Ding et al. 2025).

Commercial phage products for *Pseudomonas* do not achieve the same level of consolidation as those for other pathogens. Despite growing interest in phage therapy against this strain, application outside the human context remains restricted to experimental laboratory studies. In vivo trials in animal models demonstrate efficacy against skin, lung, and systemic infections (Yang et al. 2021), while experiments in food, water, and surfaces show potential for biocontrol (Kauppinen et al. 2021; Cristobal-Cueto et al. 2021), which were highlighted throughout the article’s discussion.

## Prospects and conclusion

When analyzing the 250 refined publications, a concentration of effort was observed in research on *Pseudomonas aeruginosa*, accounting for 191 studies (76% of the data set). However, only 53 publications (approximately 21%) reported in vivo experiments, indicating that most investigations remain restricted to laboratory or early preclinical stages. This dispersion highlights a gap between experimental efficacy and validated implementation in the real world.

The use of bacteriophages for the control of *Pseudomonas* species has demonstrated considerable potential across the healthcare, agricultural, and food industries. Analysis of recent studies indicates that organisms can reduce bacterial populations, disrupt biofilms, and mitigate diseases in both plants and animals, reinforcing their value as alternatives or complementary tools to conventional antibiotic-based strategies.

Despite the promising outcomes, most advances have been achieved at the experimental and laboratory scale, and the transition of phage-based approaches into commercially viable products remains limited. Although phage-based bioproducts are emerging across various sectors, only a limited number have advanced to commercially available formulations, while others remain in experimental or compassionate-use stages.

Key challenges include the high genetic diversity of *Pseudomonas* spp. difficulties associated with the standardization of phage cocktails and heterogeneous regulatory frameworks worldwide. In addition, production costs and lengthy development timelines further restrict the availability of approved formulations, particularly for clinical applications.

To overcome these barriers and move beyond laboratory-scale implementation, several strategic directions can be envisaged. Advances in genetic engineering enable targeted modification of bacteriophage genomes to expand host range, enhance lytic efficiency, and incorporate antimicrobial enzymes such as endolysins and depolymerases, thereby improving efficacy against biofilms and multidrug-resistant strains.

Equally important is the optimization of phage formulations and delivery systems. Approaches involving nanoparticles, hydrogels, encapsulation technologies, and related materials can enhance phage stability, bioavailability, and persistence, thereby improving their suitability for agricultural, industrial, and clinical use. For a regulatory perspective, progress will depend on the development of dedicated frameworks that acknowledge the evolutionary nature of bacteriophages, ensure product safety and consistency, stimulate innovation, and foster public and stakeholder acceptance.

Taken together, current advances in the use of bacteriophages against *Pseudomonas* highlight both their significant potential and the challenges that remain for their widespread implementation. Although biological efficacy in health, agriculture, and food is increasingly documented, the transition from experimental success to standardized, scalable implementation remains uneven. Coordinated advances in formulation technologies, regulatory standardization, and industry-specific validation under real-world conditions will be necessary. However, the convergence of biotechnology, genetic engineering, materials science, and adaptive regulatory innovation positions bacteriophages as sustainable tools for managing human and animal infections, agricultural diseases, and food contamination.

## Data Availability

No datasets were generated or analysed during the current study.
